# Electronic logbooks (e-logbooks) for the continuous assessment of medical licentiates and their medical skill development in the low-resource context of Zambia: A mixed-methods study

**DOI:** 10.3389/fmed.2022.943971

**Published:** 2022-11-23

**Authors:** Sandra Barteit, Jelena Schmidt, Mwanja Kakusa, Gardner Syakantu, Aubrey Shanzi, Yusuf Ahmed, Gregory Malunga, Klaus Blass, Jessica Nieder, Petros Andreadis, Florian Neuhann

**Affiliations:** ^1^Faculty of Medicine and University Hospital, Heidelberg Institute of Global Health (HIGH), Heidelberg University, Heidelberg, Germany; ^2^Faculty 11: Human and Health Sciences, University of Bremen, Bremen, Germany; ^3^Faculty of Medicine, Levy Mwanawasa Medical University, Lusaka, Zambia; ^4^SolidarMed Zambia, Lusaka, Zambia

**Keywords:** global health, Zambia, medical education, continuous assessment, medical skills, e-logbook, digital global health

## Abstract

**Background:**

Countries in sub-Saharan Africa continue to face insufficient health education resources and facilities, as well as a severe shortage of health care professionals. In 2019, the Levy Mwanawasa Medical University (LMMU) in Lusaka was launched to address the shortage of healthcare professionals implementing a decentralized training model utilizing selected regional and district hospitals in Zambia as training sites for various cadres. Decentralization makes it more challenging to monitor the learning process as part of continuous assessment; consequently, adequate approaches are necessary to ensure the quality and quantity of medical skills training. Electronic logbooks (e-logbooks) provide a promising tool for monitoring and evaluation of the medical training process.

**Objective:**

We designed and implemented an e-logbook for Medical Licentiate students based on an existing software system. We evaluated the feasibility of this e-logbook, its acceptability among a cohort of Medical Licentiate students and their mentors, as well as its facilitators and barriers.

**Materials and methods:**

During the course of a five-week-long clinical rotation in a training site in Kabwe, Zambia, two mentors and ten students participated in the pilot study and its evaluation. A mixed-methods approach utilized log-based usage data from the e-logbook web platform and conducted semi-structured in-depth interviews.

**Results:**

Overall, both students and mentors accepted e-logbooks as a means to monitor skills development in this context, indicating that e-logbooks are a feasible tool in this decentralized setting. Feedback pointed out that the design and software-induced terminology of the e-logbook posed usability issues. The complexity and greater time commitment (mentors used a web-based platform instead of an app) limited the e-logbook’s potential.

**Conclusion:**

We conclude that there is acceptability of monitoring medical skill development through a tablet-based e-logbook. However, the e-logbook in its current form (based on an existing software system, with limited adaptation possibilities to the local context) was insufficient for the LMMU environment. Given that this was attributable to design flaws rather than technology issues or rejection of the e-logbook as a quality assessment tool in and of itself, we propose that the e-logbook be implemented in a co-design approach to better reflect the needs of students and mentors.

## Introduction

Zambia faces a critical shortage of health workers, with only 1.2 physicians, nurses, and midwives per 1,000 people (1), and a skewed distribution of these toward urban areas (2). To address the shortage of health workers, Levy Mwanawasa Medical University (LMMU), a 7,000-capacity public medical university in Zambia’s capital city Lusaka, was established in 2019. It integrated regional and selected district hospitals as training facilities for various programs that make use of existing medical infrastructure.

### Logbooks and electronic logbooks for medical skills development

To strengthen face-to-face teaching, LMMU, along with local and international partners cooperated to leverage digital developments and their potential to strengthen the quantity and quality of medical training in Zambia. As part of this collaboration, we identified the transition from paper-based logbooks to electronic logbooks (e-logbooks) as an opportunity to improve the continuous assessment of students, recognizing that the decentralized setting with hospitals hundreds of kilometers from the central campus presents challenges beyond the capabilities of the paper-based logbook to monitor and manage the skills training process adequately. Logbooks, regardless of the format, allow students to record and track procedures learned and performed, to document students’ curriculum progression, which includes a range of skills and practices to be observed and practiced during clinical rotations, and to ensure continuity of care in a facility or community (3). Mentors monitor students’ skill progressions, provide feedback and mentoring, and evaluate that students have attained the curriculum-specified level of skill proficiency. With the paper-based logbook, program management manually reviews the logbook to confirm that the student has met all curriculum-related skill requirements and issue a grade based on the logbook’s documentation. To ensure that students are exposed to the required list of skills and medical procedures monitoring their progress during practical training is particularly important; even more so in low-resource contexts where graduates may take on extra roles beyond their curriculum, such as managerial roles in rural health clinics (4). Shifting to a digital approach for continuous assessment may increase the transparency of student monitoring and evaluation during clinical training periods, as well as reduce the administrative effort of monitoring student progress by digitalizing data collection and analysis and reducing the mentors time investment in evaluating student progress (3, 5). Additionally, the obtained data may be used in a broader context to assess clinical training and teaching, whether or not specific operations can be performed in adequate numbers in a given institution (5), or by providing valuable insights that might contribute to curriculum improvements (3). There are few studies on the use of e-logbooks in low- and middle-income countries (LMICs) (6–10); most e-logbooks were developed in high-income countries (HICs) (11, 12). In LMICs, adoption of e-logbooks is limited due to a variety of factors including limited technological and logistical resources or a low number of technical staff (12, 13).

### Strengthening rural health care in Zambia: Medical licentiates/Bachelor of Science in Clinical Sciences

This pilot project was carried out with a cohort of Medical Licentiate students (MLs) during their internal medicine rotation. The Medical Licentiate programme was established in Zambia in 2002 as a means of task-shifting to compensate for a shortage of qualified doctors in rural Zambia. Initially, the program targeted clinical officers who already had a diploma in medicine and were being taught to achieve an advanced diploma. To facilitate job advancement, the original cohort was granted a bridge to get a degree. In 2017, the program has been offered as a degree to direct entrants consisting of a four-year curriculum including clinical practicums in the third and fourth years (14). According to the Zambia Qualifications Authority’s (ZAQA) Level Descriptors for the Zambia Qualifications Framework, a Bachelor of Science in Clinical Sciences is being awarded (Zambian qualifications framework level 7) (15). MLs receive medical training allowing them to take over some physician roles such as performing surgery, C-sections, and prescribing therapies, oftentimes even taking a broader range of tasks in the local health facilities (14). At LMMU, currently, the program admits approximately 170 ML students per year. As an integral part of their undergraduate education, ML students complete two years of clinical rotations in provincial or district hospitals, where they develop critical competences in four important specialties – obstetrics and gynecology, pediatrics, surgery, and internal medicine (16–18).

### Medical licentiate programme: Current practice

**FIGURE 1:**
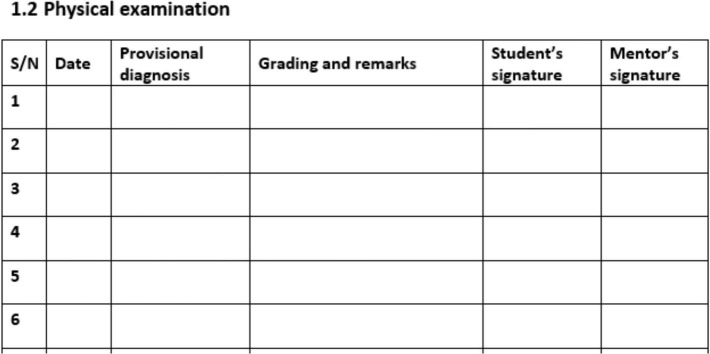
An exemplary page out of the ML paper-based logbook, showing the provision to provide a date, provisional diagnosis, grading and remarks, student’s signature, and mentor’s signature.

As students observe, assist, or do clinical activities, they record their progress in a paper logbook and a mentor ensures correctness of recorded training activities. The recording of students’ medical activities is the primary objective of a logbook. The paper-based logbook, which must be signed by both the student and the mentor at the end of the clinical rotation, serves as a record of the student’s completion of required medical procedures. The logbook is then collected and manually evaluated by LMMU staff at the end of the semester, with mentors’ evaluations having to be entered into a spreadsheet by scanning handwritten notes, which is a very lengthy procedure. Forty percent of the final grade is based on the continuous skills assessment as part of the practicum rotations, with the majority of this weighted toward theory and OSCE (objective structured clinical examination) examinations at the end of rotation. Currently, ward work assessments, as recorded in the logbook, contribute 5% toward the final academic grade. However, in its current format and use, the logbook did not serve the purpose of providing continuous assessment. According to an internal evaluation, the process for approving logbook-based procedures was not regularly performed. For example, during a previous clinical rotation, some students’ paper-based logbooks went missing, preventing them from being evaluated for their final grade. Also, the physical space in the logbook for the key aspect of feedback from mentors and grades, was very restricted. In the paper-based logbook, there was a single line for recording the date, the tentative diagnosis, and the mentor’s comments and grades (see Figure 1). The diagnosis had to be accommodated in a space of one or two brief words, the constructive feedback in two or three words. Typical comments were “Okay,” “Good job” and “Fair effort.” Mentors frequently graded and commented on procedures documented by students in the logbook without adequate verification, often with identical comments. E-logbooks provide transparent monitoring and assessment of the medical training process, making it easier for mentors and study program management to monitor each individual student’s progress and evaluate if they are on track to attain the required curriculum-based skill competency. In addition, the date and time are automatically recorded in an e-logbook, which may increase motivation to conduct the evaluation immediately after the students’ record the medical procedure.

To address the challenges outlined by the paper-based logbook and leverage the already implemented e-learning infrastructure (server, tablets distributed to students and mentors) at LMMU (16–18), local and international partners investigated how digital technologies may address some of these difficulties through the development of an e-logbook for continuous assessment of medical skills training of MLs. The primary objectives of this pilot study were to evaluate the acceptability and feasibility of the e-logbook in improving skills-based training for ML students at LMMU. In particular, the study was guided by the following overarching research questions:

(i) Is the e-logbook a feasible tool for strengthening evaluation and continuous assessment of the medical training of ML students?
(ii) What are barriers of the e-logbook within this context?
(iii) What are facilitators of the e-logbook within this context?
(iv) Is the e-logbook accepted by students and mentors?

## Materials and methods

### Study design

**FIGURE 2:**
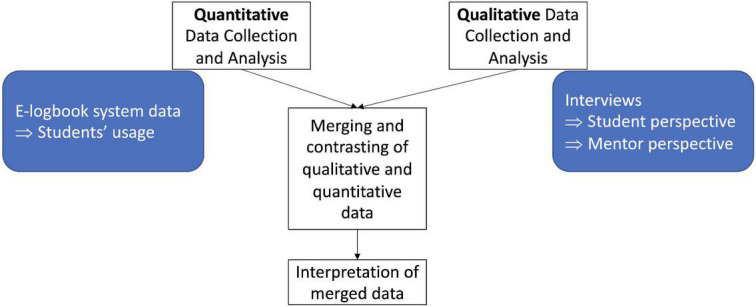
Overview of convergent mixed-methods design, in which quantitative and qualitative findings were combined and contrasted to achieve a holistic understanding of the collected data. Quantitative data collection included data from the e-logbook system, i.e., student usage data; qualitative data collection included data collected through in-depth interviews with students and mentors.

The study employed a convergent mixed-methods methodology, incorporating qualitative data from semi-structured in-depth interviews (IDIs) with students and mentors and quantitative observational data from the e-logbooks’ system data (see Figure 2) (19). Following data collection, the findings were merged and contrasted to determine the extent to which the results converge or diverge. The Consolidated criteria for Reporting Qualitative research (COREQ) (20) and Strengthening the Reporting of Observational Studies in Epidemiology (STROBE) (21) guidelines were followed.

### Study procedures

Participants were invited to an orientation session in July 2020, where they were given access to the e-logbook and instructed on its use by LMMU IT staff, who also provided support via phone or text messages throughout the pilot phase. In particular, ML students were instructed to use the e-logbook to document their clinical activities during their clinical rotation, and mentors were instructed on how to access and review individual e-logbooks and provide feedback to ML students via the web-based e-logbook platform (online).

At the end of the five week pilot, students and mentors were invited via email and encouraged to participate in semi-structured IDIs. The principal researcher (JS) conducted the in-depth interviews (IDIs) online with the use of a semi-structured interview guide.

#### Content setup

The e-logbook was translated from the paper-based version through an iterative process including content experts that included identifying adequate content structures for the e-logbook, as well as adding and updating criteria outlined in the new ML curriculum. Furthermore, the e-logbook implemented a revised and more appropriate reporting of documentation of patient cases, and observed and performed procedures, as compared to the paper-based logbook.

#### Technical setup

We developed an e-logbook by adopting the World Bank’s open access software Survey Solutions to house the e-logbook (22). Survey Solutions is a tool originally developed to conduct population-health surveys. A key advantage of Survey Solutions is that it provides an online application (app) that can be downloaded onto mobile devices and tablets facilitating mobile use. Furthermore, an internet connection is only needed to download the app and to synchronize data with the central server when new information is entered in the e-logbook, otherwise the app can be used offline. This component is critical for usage in an environment with an intermittent internet connection. Furthermore, the software has an online comprehensive monitoring component that allows mentors to track student progress and activities (Survey Solutions Headquarters), however it is only accessible through the web-based platform.

The ML e-logbook infrastructure was set up locally on the LMMU server. All study participants received tablets as part of their ML curriculum, on which the e-logbook app was installed. Onsite trainings for students, mentors and IT staff were provided by consultants who were experts in their respective fields, Survey Solutions and e-logbook for continuous assessment of medical skills training, respectively.

#### E-logbook procedures

**FIGURE 3:**
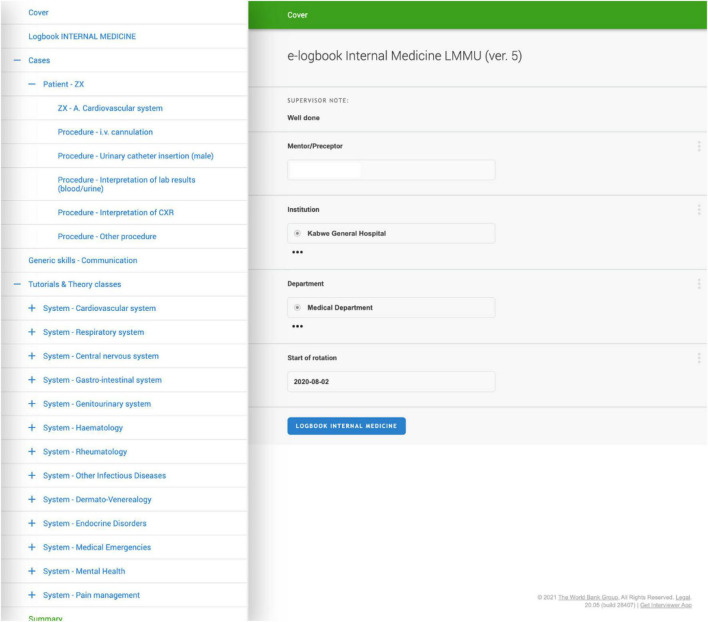
E-logbook for the internal medicine practical rotation showing the first page of a case report of patient ZX, whereby the student performed procedures in intravenous cannulation, urinary catheter insertion, interpretation of lab results, interpretation of chest radiograph and another procedure. On the left is an illustration of the e-logbook’s content structure.

As part of their e-logbook documentation, students first provide information on age, gender, and affected organ systems of the patient. Medical procedures can be documented with up to seven distinct activities and a self-evaluation of performance. In addition, students pick from a number of probable diagnoses and evaluate their own diagnostic skills (see Figure 3). After submitting the e-logbook to the server for review by the mentor (through a button click), the e-logbook is stored on the server and the student has no access to the e-logbook until the mentor sends back the e-logbook with an evaluation. As part of the pilot, students and mentors reached an agreement on the frequency of e-logbook submissions; this procedure will be formalized for the full implementation. This approach of not having access to the e-logbook while the mentor was reviewing the e-logbook was implemented for two reasons: first, to facilitate the transition from paper-based to digital processes as part of the e-logbook by replicating the physical paper-based e-logbook process, and second, due to limitations imposed by the Survey Solutions software underlying the e-logbook.

To start an e-logbook review, the mentor must review all server-stored e-logbooks and identify the relevant e-logbook (usually a logbook requires assessments from multiple mentors). After evaluating the student’s performance, the mentor will digitally return the e-logbook to the student. The mentor’s evaluation consists of constructive feedback and a grade from five defined grading categories ranging from “excellent,” “comprehensive,” “confident” and “correct” to “incomplete,” “more serious oversights or errors,” “need for repetition.”

### Study participants and study duration

During a five week internal medicine rotation at Kabwe General Hospital from August 3rd to September 4th, 2020, *n* = 10 ML students and *n* = 2 mentors participated in the e-logbook pilot study. The sampling procedure was purposive and non-random as all ML students and their mentors at Kabwe General Hospital involved with the e-logbook were included. Interviews with *n* = 5 students and *n* = 2 mentors were deemed sufficient to achieve information saturation.

Only students and mentors who consented in written form to take part in the study at the introductory workshop were eligible. Each research participant was assigned a code (S1, S2) to pseudonymize participant information.

### Study setting

Kabwe Central Hospital is in Zambia’s central province, around 140 kilometers north of the country’s capital, Lusaka, providing second-level health care services to nine districts. It is a hospital where ML students complete a five week practical rotation in internal medicine. Besides ML students, Kabwe Central Hospital also supervises further healthcare professional students from private and public educational institutions, including medical students, nurses and others.

### Data collection and analysis

#### Quantitative research component

At the end of the study period, quantitative data on student use of e-logbooks was downloaded from the e-logbook web platform based on Survey Solutions. Throughout the study period, students transferred data to the web platform by syncing the e-logbook app on their tablets online, when an internet connection was available. Students had to manually synchronize the e-logbook by clicking a button; otherwise, the e-logbook and its entries were unavailable to mentors. Mentors, on the other hand, were required to log in to the web platform, as Survey Solutions did not offer an app for managing submitted e-logbooks. Collected data included whether or not students accessed the e-logbook, the number of patient cases and medical procedures performed, whether or not the e-logbook was submitted to the mentor for review, and whether or not the e-logbook was returned to the student with feedback. Relevant data (created between August 3rd to September 4th, 2020) were exported in MS Excel format, and descriptive analyses were performed to assess individual usage of the e-logbook.

#### Qualitative research component

**FIGURE 4:**
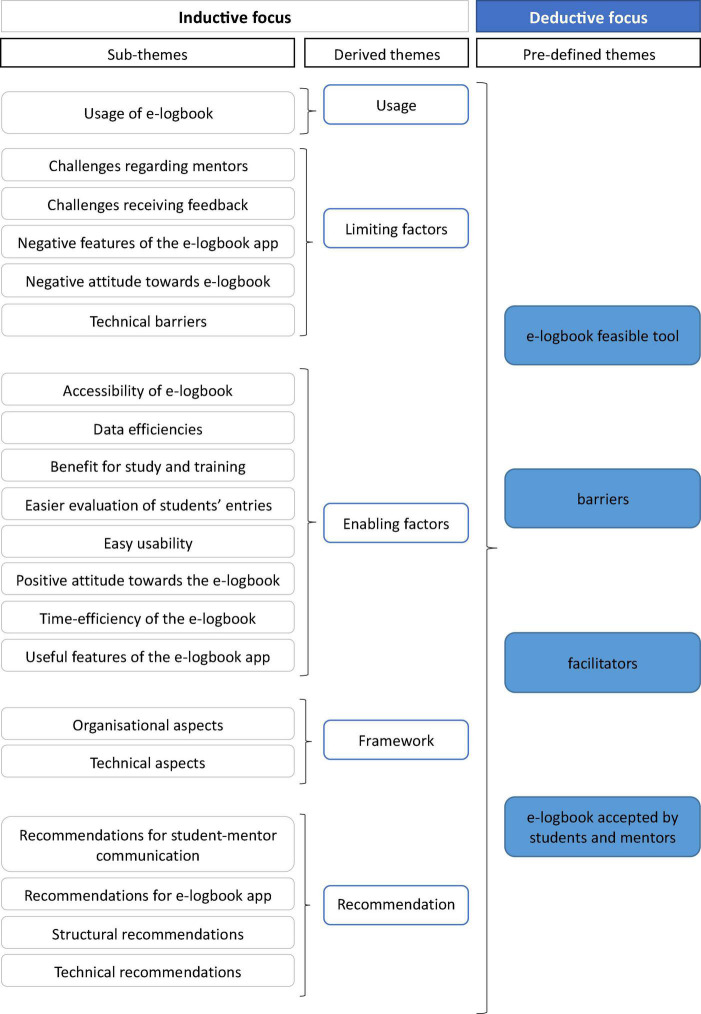
Overview of methodological approach, showing the inductive and deductive process.

The IDIs were carried out to understand students’ and mentors’ perspectives and experiences with the e-logbook, as well as their acceptance. The IDIs were digitally recorded and transcribed using the software MAXQDA.^1^ Summative qualitative content analysis was used to systematically analyze interview transcripts and identify emerging themes in IDIs with ML students and mentors, as described by Mayring (23). In a first step, deductive analysis was used to categorize responses into four pre-defined themes that were constructed to address the four research questions and allow for comparison between interviewees. Following that, sub-themes within the four preset themes were developed using an inductive technique (see Figure 4). This resulted in five new themes, which were discussed and agreed upon by the research team. The five themes were (1) usage, (2) limiting factors, (3) enabling factors, (4) framework, and (5) recommendations.

## Results

A total of seven interviews (*n* = 5 students, out of a total of 10 students; *n* = 2 mentors, out of a total of 5 mentors) were conducted between 12th September, 2020 and 23rd October, 2020. The interviews were conducted via online platforms and the interviewees were assigned codes: S1, S2, S4, S5, S9. In the following result section, we detail the interviews with specific reference to the study participants and the lines of the transcription, i.e., S1: 78–82. Interviews were indicated as I1, I2, etc. (see Table 1).

**TABLE 1:** Overview of assigned categories and respective interviews with their identifier.

Theme	Sub-theme	Interview
Usage of the e-logbook	Usage of the e-logbook	I1, I2, I4, I5
Factors limiting the use of e-logbook	Challenges concerning the mentors	I1, I2, I5
	Challenges in receiving feedback	I1, I2, I3, I4, I5
	Negative features of the application	I1, I2
	Negative attitude toward the e-logbook	I1, I2
	Technical barriers	I1, I2, I3, I4, I5
Factors enabling the use of the e-logbook	Accessibility of e-logbook	I2
	Central saving of data	I3
	Benefit for study and training	I1, I3
	Easier evaluation of students’ entries	I3, I4
	Easy usability	I1, I2, I3, I4, I5
	Positive attitude toward the e-logbook	I1, I2, I3, I4, I5
	Time-efficiency of the e-logbook	I1, I2
	Useful features of the application	I2, I3
Framework	Organizational factors	I5
	Technical support	I1, I2, I3, I4, I5
Recommendations	Recommendations concerning the student-mentor communication	I1, I2, I5
	Structural recommendations	I5
	Recommendations for the application	I1, I3
	Technical recommendations	I1, I2, I3, I4

### Interviews (Qualitative evaluation)

Two mentors and five ML students took part in the IDIs. Nineteen sub-themes were generated from the content analysis of the students’ interviews, while 14 sub-themes arose from the content analysis of the mentors’ interviews. These were categorized into five themes regarding the e-logbook: (1) usage, (2) limiting factors, (3) enabling factors, (4) framework, and (5) recommendations.

In the following, the main IDIs results are presented under the five new themes developed during the inductive process.

#### E-logbook usage

One student described the usage of the e-logbook as following: “*I go on the ward or maybe in the outpatient department. I clerk my patient then I’ll get that information and feed into the system. When I am done with [*…*] that interview, then I will send it to the consultant.*” (S5: 213–216). All interviewees reported their progress throughout their practical rotation using a smartphone or a tablet on a regular basis. None of the interviewee reported usage difficulties. In general, the usage of the e-logbook was hindered by technical issues, notably a reliable internet connection for data synchronization. However, ML students reported that the Wi-Fi network was frequently unavailable and difficult to connect to. Students therefore used their phones as mobile hotspots to link their tablets to the internet. Mainly, students used the tablets they have been provided through LMMU to access the e-logbook app. As the tablets handed to students as part of their ML training did not support the usage of a SIM card, students felt their capacity to connect their device to the internet was greatly constrained, hence restricting their use of the e-logbooks: “*the tablets have got no sim card, so you really have to rely on a hotspot from the other phone like to send you interviews for the patient and stuff so that it is really proving some kind of a challenge*” (I3: 3–7). Students suggested better internet connectivity to improve accessibility to the e-logbooks.

#### Limiting factors

Overall, the introductory workshop which provided onsite training was perceived to be helpful in supporting the use of the e-logbook. Nevertheless, both, students and mentors, initially had reservations about the e-logbook. Students were especially concerned whether they were able to get a fair rating as they were being rated on their clinical training asynchronously by mentors (S1: 78—-82): “*thing that actually made it sceptical was like […] am I going to be able to get a fair rating that was the concern*.”

Overall, the e-logbook was intended to facilitate communication between students and mentors and allow for a continuous assessment of students. All students interviewed expressed difficulty receiving feedback on their e-logbooks, as one student reported “*the feedback is not given there and then*” (S1: 56–57). Instead, students had to wait for feedback from the mentors for some time and did not receive feedback on a continuous basis throughout the rotation. During the study period, mentors were supervising not just ML students but also students pursuing other healthcare professional degrees. This double burden was reflected on by a student: “*so in terms of maybe giving feedback at the end of the day it is quite challenging for them also as you can imagine maybe you have 10 students then all of them feedback maybe five patients per day and then they off send this information to the consultant who is been working the whole day you can imagine*. *[…] he or she has to go through maybe 50*” (I5: 182–188). During the interviews, it became apparent that students were submitting their e-logbooks to mentors who were not in the hospital with them every day, that hindered the feedback process, and therefore students recommended: “*assessed by the person […] who is working with us right now*. *[…] that person is able to provide feedback quickly*. *It would be more motivating*” (I1: 117–120.).

Also, there was confusion regarding the review turnover, as one student commented that the e-logbook should be handed in for review twice a week, which contradicted the mentors’ perspective, as reported in the following: “*the agreement with the students was that during the week they would do the work. And then submit at the end of the week*. *(…) So that we [the mentors] can work on the logbook over the weekend*” (I1: 15–16). Mentors reported that they struggled with their feedback turnover: “…*and then they only hand in their logbooks at the end of the week, right? Or which I may have forgotten what I said on the Monday*. *So, I have to now start remembering and then trying to correlate with what is going on now*. *You get things mixed up*” (I1: 64–67). Furthermore, one mentor reported confusion due to multiple e-logbook versions present in the system from one student. In general, mentors perceived the e-logbook as time-demanding and difficult to use. The two main reasons were: (1) the terminology stemming from Survey Solutions, which did not well adapt to the medical context in Zambia (and could not be adjusted, as Survey Solutions has no provision for changing terminology), (2) learning how to navigate the new e-logbook: “*is it not very encouraging, if you have to spend so much time on a logbook and you look at how much work is involved with the number of students that we look after, as well as other responsibilities within the hospital setup*” (I2: 89–92).

#### Enabling factors

Students perceived the e-logbook as an easy and time-efficient tool: “When you are using it you don’t have to write something, you just have to click, it opens, you just fill in the information.” (S2: 30–32). “you log in you just log out” (S2: 85–86) and “you have enough time (…) to do other things.” (S2: 144–146). In addition, it was mentioned in the interviews that paper-based logbooks were sometimes only filled out when the central administration needed them for evaluation, whereas the e-logbook encouraged continuous documentation of medical skills training: “you don’t have to go back and sit on the table after signing start again those things you know so it is faster.” (I1: 85–90). Another student mentioned: “there are a lot of options […] which you can choose and anyway you can choose you specify, so I think according to me […] you benefit more if you use the electronic logbook than the paper based on” (I2: 184–187).

During clinical rotations, the e-logbook was seen very beneficial since students did not have to wait for the mentor present in the hospital to sign it; instead, they could independently fill out and send the e-logbook to their principal mentor, which was not possible with the paper-based e-logbook.

The fact that the e-logbook is stored electronically on the tablet and server, which prevents data loss, was seen as a benefit by students: “*it is accessible everywhere [*…*] and it can be done anytime*” (S2: 159–160).” Particularly, as students had reported in interviews that paper-based logbooks had gone missing during a previous clinical rotation and could be retrieved for their final evaluation: “*there are no other giving excuses of saying I misplaced the logbooks and will not send them school for your final evaluation when you are at your final exams that thing won’t be there because it will be in the database*” (S3: 84–87).

Furthermore, mentors felt the updated and expanded content of e-logbooks to be beneficial: “*the expected competencies in terms of the procedures and skills*. *It is all clearly outlined and I think readily available for the students and the lecturers [mentors] or the preceptors to look at and see what has been covered and what is still missing*” (L2: 230–233).

#### Framework

One student remarked that the clinical rotation was disorganized because their group was not informed of the need to do this evaluation until they arrived at the rotation site: “*I haven’t been using much like the tablet*. *So, when I came from Kabwe to Kabwe, I left it at home*” (S5: 11–12).

It should be noted that the pilot phase took place during the COVID-19 pandemic, which drastically altered and impacted clinical training. One student mentioned this as well: “*there was a lot of confusion because of the corona issues*” (S5: 103).

#### Recommendations

Mentors recommended students to fill out their comments in the e-logbook immediately after performing clinical procedures: “… *the easiest thing would be, the student has a tablet, they type what they have done and then just sign, right there [*…*]*” (L1: 100–102) – which is consistent with the students’ desire for feedback from the mentor present during the clinical procedure. Furthermore, mentors recommended the e-logbook’s user interface be improved to save time searching for specific students’ e-logbooks.

Students proposed adding more categories to the e-logbook that reflect various medical practises and clinical activities to better represent the daily scope in practicum sites, as well as a free text option to record lengthy diagnoses or co-morbidities. Furthermore, they emphasized the significance of readily available and accessible internet, as well as tablets with SIM card slots to avoid relying on other sources for internet access.

### Usage data (Quantitative evaluation)

Eight students downloaded their e-logbook on their tablet, one student used a smartphone, and one student never downloaded their e-logbook. Seven of the nine students who downloaded their e-logbook onto their tablets were active users, which means they submitted their completed e-logbook to their mentor at least once throughout the research period. The number of documented patient cases per student ranged from 1 to 14, while the number of medical procedures performed per student ranged from 2 to 35.

Two mentors were responsible for the students’ e-logbooks and respective feedback during the pilot phase; each mentor reviewed one e-logbook comprising recorded patient cases and medical procedures performed by students. Five e-logbooks did not receive a review. One of the reviewed e-logbooks was returned to the student, while the other was sent to the server for final assessment, meaning that the student no longer had access to the e-logbook.

During the pilot phase, all students who had their e-logbooks evaluated sent them to their mentor once. However, none of the students completed the task of sending 10 e-logbooks over the course of the study period of five weeks. We found that each student documented at least one patient per e-logbook with a minimum of two performed medical procedures.

## Discussion

The evaluation of the pilot study yielded insights into the barriers and facilitators of the e-logbook for the ML program of the LMMU in Zambia. Overall, we found an e-logbook to be a feasible tool. Students and mentors reported general acceptance of the e-logbook as a tool for monitoring and evaluating medical skill development during the clinical rotation; however, we observed challenges with the current e-logbook design that necessitate reconsidering the current usage flow and re-designing another version of the e-logbook based on the feedback received as part of this pilot study.

Students perceived the e-logbook positively, highlighting its ease of use and potential benefit to their practical training. Our findings confirm prior research that has found that students indeed prefer e-logbooks (8). The fact that students expressed high perceived ease of use and usefulness is especially encouraging, as these variables are positively associated with the intention to use digital technologies, such as an e-logbook (24, 25). Further supporting this argument, Viseskul et al. found in their study also a high degree of satisfaction with the e-logbook, which students rated as a valuable tool for analysing and tracking their academic performance (8).

Nevertheless, the introduction of the e-logbook evidently induced a new workflow for both students and mentors, which also resulted in the perception of a new digital burden that involves an additional time investment since neither students nor mentors were habituated to the e-logbook app and its inherent processes. In addition, the lack of an app to provide more convenient access for mentors to manage submitted e-logbooks – they had to log in to a web platform – the absence of financial incentives associated with e-logbooks (the e-logbook may have been perceived as an extra activity which may have created expectations for additional financial compensation), the overburdening of student mentorships at the study facility, and insecurity with digital tools appeared to be the factors that adversely affected participation in the e-logbook based continuous assessment. To promote acceptance, (continuous) training can play a key role in overcoming barriers and increase ease of use (26), as well as elements of co-designing and translating learnings from this study for a potential new version of the e-logbook, that is better tailored to the needs of this specific context. In the future, IT staff might potentially offer regular e-logbook trainings for students at the LMMU main campus in Lusaka, as well as comprehensive and targeted trainings for mentors on location at the hospital or health institution. Considering that e-logbooks are a new digital tool for students and mentors, it necessitates a change of practice and habits (26). Overall, students and mentors reported no difficulties in using digital devices on a regular basis; however, because these statements were based on self-evaluations, they may have been influenced by social desirability, emphasizing continuous training as a key factor boosting adoption of new digital technologies and overall digital literacy (27).

Nevertheless, e-logbook adoption was limited during the pilot phase, which may be due to the short study period of five weeks, which made it difficult to implement change processes and provide sufficient training for students and mentors to fully adopt the e-logbook. For example, mentors failing to provide timely feedback, making it difficult for students to document new procedures in the e-logbook because the e-logbook was still checked out by mentors and not accessible to students.

Mentors also reported more barriers with using the logbook. The introduction of new digital interventions may have put additional strain on already overburdened medical professionals who do not have the capacity to work through technical challenges (28). Mentors are often double-burdened since they are involved in both clinical work as medical professionals and mentoring students – which is a result of the human resource shortages in Zambia. Therefore, perceived usefulness emerged as a key factor in the IDIs as mentors reported additional time-burden to locate and review each e-logbook, which impeded adoption of e-logbook (25). To better prepare mentors for the e-logbook use and facilitate their mentorship activities, we would foster training sessions potentially on a continuous basis, peer assistance, or social learning (29, 30).

Another hindering factor of this e-logbook was its usage flow and terminology. Nonetheless, mentors noted the e-logbooks’ potential benefits, such as the ease of determining which medical procedures students still needed to perform, emphasizing that mentors were also recognizing positive aspects of the e-logbook.

Technical issues were not a dominant theme; rather, we found that the usage flow and content structure of the e-logbook hampered uptake. Furthermore, our findings highlight the importance of the terminology of the e-logbook to reflect day-to-day terminology used in the clinical context of Zambia. Since it is impossible to modify terminology embedded in the Survey Solutions software, for example, “reject interview” in the e-logbook referred to sending the feedback provided by the mentor back to the student, we think it to be more constructive to design an e-logbook from scratch that can reflect the needs of the Zambian context. It seems advisable to design and build an e-logbook following a bottom-up-approach, leveraging co-design components and design thinking approaches including a staged roll-out and an evaluation. A self-developed version has the added benefit of greater operational flexibility, such as allowing students access to the e-logbook throughout the mentor review process. One disadvantage of the self-development of an e-logbook is that maintenance expenditures will be incurred, as the software will need to be updated eventually to meet the operating system requirements of the tablets, among other things. The fact that the software for the ML e-logbook is maintained by the World Bank, which publishes regular updates and thereby increases overall sustainability in a low-resource context and reduces the need to employ software developers to maintain the software, was an initial deciding factor for using Survey Solutions. Overall, open-source solutions appear more cost-effective than self-developing software, which was not an option for us, but could be in other contexts. Resultantly, we will develop an e-logbook software, which we hope to release as an open-source project so that others in similar contexts may use it and build upon it. We did not investigate subscription-based software solutions because we do not consider them practical or tenable in a low-resource setting.

According to our experiences, incorporating tablets in a low-resource context to support medical education in a blended learning approach has been well received by students, faculty, and mentors (17, 18). Students were able to access and input data even if not connected to the internet, and data was synchronized once the tablet was connected to the internet. The institutionalization of tablets and their financial management, particularly in terms of procurement and maintenance, has proven to be a significant obstacle, which still requires a solution.

The e-logbook proved useful in the sense that the technical setup worked in this setting and provided constant access to the e-logbook technically, as well the continuous synchronization of data to the main e-logbook server prevented e-logbooks from being lost – as was the case in the past for the paper-based e-logbooks. Once in place, the e-logbook is expected to decrease administrative time and effort because data for each student is digitally available and does not need to be assessed manually. Managing paper-based logbooks for decentralized training programs like those offered at LMMU becomes increasingly onerous as the number of students at LMMU is projected to further increase in the next years to come. The primary barriers to utilizing e-logbooks were interface issues such as terminology and the e-logbook being seen as too demanding and time consuming by mentors. The majority of these issues were attributable to the software system that did not permit better customization of the e-logbook.

### Limitations

There are various limitations to the study. Firstly, the study population consisted of just a small number of students and mentors, limiting generalization. Secondly, rather than being an impartial researcher, the interviewer was participating in the study, which may have influenced participant attitudes. To reduce the impact, interviews were done by a researcher who had no prior ties to any of the individuals. Thirdly, rather than being developed by a team, the themes were generated by a single researcher, which may have resulted in subjective interpretation. However, we found the results of this smaller pilot of the e-logbook to be appropriately informative. As the study population for this pilot study was rather small, quantitative results may lack significant scope (statistical power); nonetheless, we view this as a minor constraint as quantitative data were only utilized to validate reported usage.

## Conclusion

The purpose of this study was to evaluate the feasibility and acceptability of an e-logbook for continuous evaluation and monitoring of the MLs’ medical skills training at LMMU in Zambia. We found that the e-logbook was largely accepted by students and mentors and that the e-logbook as a digital tool is feasible for the evaluation and monitoring of skills training offering the potential to strengthen medical skills training in such a low-resource setting. Despite its pilot character, our study shows that while new digital technologies can be valuable, integrating them in complex environments such as hospitals may require continuous training and efforts, so that these systems can be effectively integrated into daily work routines so that their benefits can be fully realized. As a next step, we will focus on a co-designed and context-specific e-logbook to become a beneficial tool for mentors and students. In general, it is essential to limit the disruption that digital innovations may cause by ensuring that everyone involved is adequately trained on how to use new digital tools, as well as the digital tools need to be context-relevant, to avoid digital fatigue which may impede acceptance and adoption of potential digital solutions.

## Data availability statement

The raw data supporting the conclusions of this article will be made available by the corresponding author upon request.

## Ethics statement

This work was authorized by the Research Ethics Committees of Levy Mwanawasa Medical University and Heidelberg University Hospital (S-258/2021). All participants were informed about the study procedures and provided written informed consent for their log data to be recorded and analyzed, as well as their participation in the IDIs.

## Author contributions

JS, SB, PA, and FN developed the objective and research question. JS conducted qualitative and quantitative data analysis. FN developed and coordinated the lists of procedures. KB conducted the technical setup of the e-logbook in Survey Solutions. KB and GM conducted e-logbook trainings and provided technical e-logbook support. JS conducted data extraction, analysis, and interpretation with support from SB. JS and SB wrote the manuscript draft. PA and FN contributed extensively to the further manuscript revision. All authors approved the final version of the manuscript.

## Footnotes

1
https://www.maxqda.com/
